# Characterization of Chickpea (*Cicer arietinum* L.) Flour Films: Effects of pH and Plasticizer Concentration

**DOI:** 10.3390/ijms20051246

**Published:** 2019-03-12

**Authors:** Olga Díaz, Tania Ferreiro, José Luis Rodríguez-Otero, Ángel Cobos

**Affiliations:** 1 Área de Tecnología de Alimentos, Departamento de Química Analítica, Nutrición y Bromatología, Facultad de Ciencias, Universidade de Santiago de Compostela, 27002 Lugo, Spain; tania.ferreiro@usc.es (T.F.); angel.cobos@usc.es (Á.C.); 2 Área de Nutrición y Bromatología, Departamento de Química Analítica, Nutrición y Bromatología, Facultad de Ciencias, Universidade de Santiago de Compostela, 27002 Lugo, Spain; jlr.otero@usc.es

**Keywords:** edible films, mechanical properties, microstructure

## Abstract

The use of flours as a material for biopolymer-based film preparation has gained interest due to the fact that they are a natural mixture of compatible macromolecules and due to their low cost. Chickpea flour shows a promising composition for the development of edible films. The aim of this study was to characterize and evaluate the properties of chickpea flour films as affected by pH (7 or 10) and plasticizer concentration (1% or 3% *w*/*v*) of film-forming solutions. Water vapor permeability, solubility, color, opacity, mechanical properties, thermal stability, structural changes by Fourier transform infrared analysis, and microstructure of the films were determined. Glycerol content and pH influenced chickpea flour film properties, microstructure and structural organization; interactions were also observed. The 1% glycerol films showed lower water vapor permeability, thickness, radical scavenging capacity, elongation at break and puncture deformation, and higher dry matter content, swelling, opacity, elastic modulus, and tensile and puncture strengths than 3% glycerol films. Film-forming solutions at pH 10 produced films with higher thickness and swelling, and were greener than those from solutions at neutral pH. The changes were more intense in 1% glycerol films. Glycerol concentration and pH could be combined in order to obtain chickpea flour films with different properties according to different food packaging requirements.

## 1. Introduction

Biopolymer-based films have been widely investigated as an eco-friendly option to petroleum-based plastic materials for food packaging. They are derived from renewable natural sources and are commonly based on carbohydrates and proteins. Often, these hydrocolloids form films that show some drawbacks related to barrier or mechanical properties. For this reason, they have been combined with each other or with other compounds, such as lipids [1].

Recently, the use of flours for film preparation has gained importance due to the fact that they are natural and compatible mixtures of different components that can be easily obtained at lower cost than the mixtures of purified individual macromolecules. Films of flours from amaranth, red rice, achira, banana, quinoa, chia and pinhâo [2,3,4,5,6,7,8] have been produced and evaluated. Legume flours are also a good source of material for film formation due to their high content of starch and protein; some of them also contain a significant amount of lipids. Lentil, defatted soy and grass pea flours have been studied as film-forming materials [9,10,11]. Chickpea (*Cicer arietinum* L.) is a legume grown and consumed in more than fifty countries on five continents due to its nutritional value. It is a good source of proteins and carbohydrates, including dietary fiber, and, although it cannot be classified as an oilseed crop, chickpea lipid content is higher than that of other pulses [12]. Chickpea proteins are mainly globulins, followed by albumin, and small amounts of glutelin and prolamin. Their solubility is low, close their isoelectric point (pH 4–6), and high at alkaline pH values, and their thermal denaturation starts around 65 °C for most thermolabile globulin [13,14]. Starch is the main carbohydrate (40–44% dry weight) and contains 30–35% total amylose; its mid-point gelatinization temperature is 64–73 °C in flours [15,16]. Chickpea starch and proteins exhibit good emulsifying, foaming and pasting properties [17]. Additionally, chickpea contains bioactive compounds with antioxidant potential, including peptides and phenolic compounds [18]. Accordingly, chickpea flour shows an interesting composition for the development of edible films, but it has not been studied to this point.

Plasticizers are often added to film formulations to improve film flexibility, elongation and ductility by reducing the intermolecular forces among polymer chains; the type and concentration of plasticizer also affect the thermal and barrier properties of films with a protein or starch base. Glycerol is a commonly used plasticizer due to its high efficiency, rapid polymer diffusion and interaction; other compounds such as lipids and water can also act as plasticizers [19,20].

The objective of this work was to characterize chickpea flour films by evaluating the effects of pH and glycerol (used as plasticizer) content in the water vapor permeability, solubility, antioxidant capacity, color, opacity, mechanical properties, thermal stability, and microstructure of these films. Some structural changes to flour components, induced by these factors, were also investigated by Fourier transform infrared spectroscopy. The properties of the films based on this renewable natural biomaterial can be easily modified by changing pH and glycerol content and thus could meet particular packaging demands in food applications.

## 2. Results and Discussion

### 2.1. Water Vapor Permeability, Thickness, Dry Matter Content, Solubility and Swelling

Table 1 shows the values of water vapor permeability, thickness, dry matter content and solubility of chickpea flour films.

Water vapor permeability (WVP) was significantly affected by plasticizer concentration; 3% glycerol films showed the highest values. Similar results about the influence of high glycerol concentration have been found by other authors studying starch films [19,21]. Plasticizers decrease intermolecular forces among polymer chains, increase free volume and make the film matrix less dense and, at high concentrations, enhance mobility of water molecules across the matrix [22], increasing WVP. Additionally, glycerol has a hydrophilic nature that facilitates water sorption [19,20]. At the lowest glycerol concentration, strong polymer molecule interactions, mainly starch–starch but also protein–protein, could generate a more compact structure that decreased WVP. Glycerol at low concentrations, due to its low size, could replace water and form glycerol–starch interactions that also compact the structure [21]. An antiplasticization effect could also be involved. Adhikari et al. [22] reported that low concentrations of glycerol (below 15% *w*/*w* starch basis) decreased moisture migration rates in low amylose starch films; they attributed this effect to the strong hygroscopicity and hydrophilic nature of this plasticizer, which retained water molecules and did not allow their diffusion through the film structure. In the chickpea flour films studied in the present work, the lowest glycerol concentration added was of 16.7% *w*/*w* flour basis, close to the limit value cited by these authors, so it is possible that antiplasticization could occur. However, other flour components, different from starch, could also affect glycerol behavior. The WVP values of chickpea flour 1% glycerol films (16.7% *w*/*w* flour) were lower than those found in films with close glycerol content based on achira, pinhâo, chia and red rice flours [2,3,4,8], higher than in amaranth flour films [23,24], and similar to those reported in lentil films [9]. The differences with non-legume flours could be due to the hydrocolloid composition and also to the protein and lipid content of the chickpea flour. WVP was not influenced by pH.

Significant decreases in thickness and increases in dry matter content were observed in films with a lower glycerol concentration and could be due to the development of a denser structure with increased polymer–polymer interactions and low availability of glycerol to link water molecules. Alkalization of film-forming solutions affected the film thickness, which was significantly higher at pH 10 compared to that of films from solutions at neutral pH. The isoelectric point (at which net charge is 0 and there are no repulsive forces among protein molecules) of most of chickpea proteins is 4–5 [13,14]. However, 2D electrophoresis studies have found that there are a wide variety of chickpea proteins that show differences in their isoelectric point, with values under 5 to above 6 [25]. Thus, neutral pH is relatively close to the isoelectric point, so protein interactions in the film matrix are influenced by a lower net charge in which repulsion forces are reduced. This fact could decrease film thickness. At alkaline pH, the increase of negatively charged groups that repeal each other could modify protein chain structure and intermolecular bonding [26]; this fact may produce thicker films. Chickpea film thickness was higher than those found in grass pea, amaranth, achira, red rice, pinhâo and lentil flour films [2,3,8,9,11,23], lower than in chia flour films [4] and similar to that of quinoa flour films [5]. Thickness was in the range of triticale, peanut and lentil protein films, and starch films [27,28]. Dry matter content only significantly increased with alkalization in 3% glycerol films, also probably due to changes in proteins that lost some of their capacity to retain water.

Solubility was higher in 1% glycerol films than in 3% glycerol films, and interactions between glycerol content and alkalization were observed. Significant differences between films at pH 7 and 10 were only found at 1% glycerol concentration. Alkalization increases starch and protein solubility [29,30,31] and these effects were only observed in films that had less glycerol molecules; in all likelihood, more chemical groups of protein and starch could be available to interact with water. Chickpea film solubility was higher than that of films based on achira, red rice, chia and lentil flours [2,4,8,9], and similar to that of amaranth and quinoa flour films [5,23].

Swelling was affected by both plasticizer concentration and pH, although interactions were not observed. The 3% glycerol films showed lower swelling capacity than the 1% glycerol films, and values increased at pH 10. Presumably, the samples with lower plasticizer content could have more chemical groups of starch and protein available for water interaction and retention. The rise of swelling in accordance with alkalization was attributed to the interactions between starch and protein [30] and to the reduction of restraining effects of amylose in the amorphous regions of starch [31]. The values were higher than those reported in red rice flour [8] but lower than other found in protein–carbohydrate mixed films [32].

### 2.2. DPPH Radical Scavenging Capacity

Films with 3% glycerol showed higher DPPH (2,2-Diphenyl-1-picrylhydrazyl) radical scavenging capacity (Table 1) than those with lower amounts of plasticizer. Interactions between glycerol concentration and pH were observed. Chickpea has antioxidant properties due to the presence of peptides and polyphenolic compounds. Several peptides with antioxidant potential have been identified from chickpea protein hydrolysates and considerable amounts of phenolic compounds and anthocyanins with antioxidant activity have also been detected in chickpea [18], although their concentration depends on the type of chickpea; the Kabuli type used for flour manufacture in Europe contains lower amounts of these compounds [33]. As the phenolic compounds of chickpea flour were not modified during film preparation, the changes in antioxidant capacity probably arose from protein modifications; however, protein–phenolic compound interactions could also be involved. The information about the effect of protein–phenolic compound complexation in the antioxidant capacity is contradictory; some authors have reported decreases [34] while other authors described increases in this property in peas [35].

High concentrations of glycerol could reduce intermolecular bonding among proteins and change their conformation, probably exposing antioxidant groups of amino acids that may increase the antioxidant capacity of 3% glycerol films. Glycerol at high concentrations interacts with polymers through hydrogen bonding, Van der Waals forces, etc. As protein–phenolic interaction mechanisms include these types of bonding, it is possible that the complexation may be less important in these films. In 1% glycerol films, polymer–polymer interactions could involve the chemical groups with antioxidant properties in amino acids, and those that could react with phenolic compounds, reducing their antioxidant capacity. The effect of alkalization was different depending on the amount of glycerol added. The antioxidant activity increased in 1% glycerol samples at pH 10, probably due to the changes in protein conformation without the interference of high amounts of plasticizer, and also to the formation of complexes of proteins with polyphenolic compounds that increased at high pH values [34]. The decrease in 3% glycerol pH 10 films might be attributable to the interaction between plasticizer and amino acid groups, exposed by the changes in protein conformation, which reduced the possibility of complexation between protein and phenolic compounds.

### 2.3. Color

Table 2 shows CIEL*a*b* color values, total color difference (ΔE) and opacity of chickpea flour films. L*, a* and b* values were similar to those reported in quinoa flour films [5].

Glycerol concentration significantly affected L*, b* and the opacity of films that were slightly darker, less yellow and more transparent, with the highest plasticizer addition. Interactions with pH were observed in a* values; they were higher at neutral pH and lower at pH 10 in 3% glycerol films, compared to 1% samples. The opacity also showed interactions and was only affected by the pH in 1% glycerol films. Differences in film structure could be responsible for these results. Changes in color due to alkalization (decrease of a*, increase of b*) could be due to the formation of complexes between proteins and polyphenolic compounds [26]. The intensification of Maillard reactions at alkaline pH could also have increased the yellowish color of films [36].

### 2.4. Mechanical Properties

Mechanical properties of chickpea flour films are shown in Table 3. Tensile strength values were in the range of those described in achira, lentil and banana flour films with similar glycerol content [2,6,9], higher than that of grass pea films [11], while elongation at break values were lower. The mechanical properties of mixed, heterogeneous systems such as flour films are a consequence of interactions among their components (protein, starch, lipids) [23], and varied with the flour origin and composition.

Tensile strength, elastic modulus and puncture strength decreased, and elongation at break and puncture deformation increased with increasing plasticizer content, as has been reported for polysaccharide and protein films [20]. High amounts of plasticizer reduced the cohesive forces between polymer chains, with the substitution of strong interactions with hydrogen bonds; secondarily, the hygroscopic nature of glycerol increased the absorption of water, that is also a film plasticizer [9]. As a consequence, films became less rigid and more flexible.

The effect of alkalization of film-forming solutions was different depending on glycerol content, due to interactions between the two factors. Tensile strength and elastic modulus increased in 1% glycerol films, making them more resistant and rigid; in 3% glycerol films, elongation at break and puncture deformation augmented and they were more flexible than at neutral pH. Changes in protein structure due to denaturation and, as a consequence, in the interactions with starch, glycerol or with other proteins could be responsible for these results. Starch crystallization in alkaline condition [31,37] could also have affected the mechanical properties of films. The tensile strength of 1% glycerol films was higher than that of vegetable protein films, and elongation at break was in the range of starch-based films of similar glycerol content, as reported in the review of Mauri et al. [27].

### 2.5. Fourier Transform Infrared Spectroscopy

**Figure 1:**
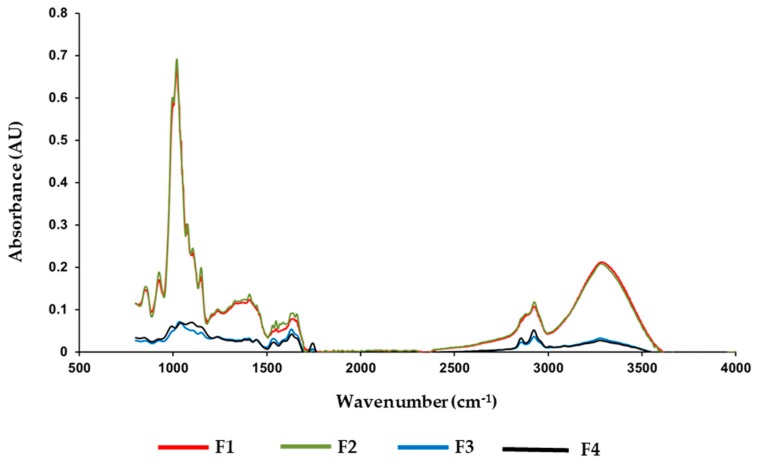
FTIR spectra of chickpea flour films. F1: 3% glycerol, pH 7; F2: 3% glycerol, pH 10; F3: 1% glycerol, pH 7; F4: 1% glycerol, pH 10.

Fourier transform infrared spectroscopy (FTIR) spectra of chickpea flour films showed typical bands in two regions (3700–2800 cm^−1^ and 1800–650 cm^−1^), associated with carbohydrates and proteins (Figure 1).

Most bands corresponded to carbohydrates, the main components of flour, but some related to proteins were also detected. Several bands in the 1800–650 cm^−1^ region were observed in all samples; 855 cm^−1^ and 925 cm^−1^ have been attributed to C–C skeletal vibrations and to the glycosidic bonds of starch, respectively [6,9,38]. The 996 cm^−1^ band has been related to the crystalline structure of starch, the intramolecular hydrogen bonding of hydroxide groups and water sensitivity [39,40]. Other two peaks were related to the structure order of starch. The 1020 cm^−1^ corresponds to the amorphous region, while the 1047 cm^−1^ band reflects the amount of ordered regions of starch [38,41,42]. Other three bands (1076, 1104 and 1148 cm^−1^) have been attributed to the stretching of the C–C, C–O and C–OH bonds of starch. An absorption band at 1237 cm^−1^ could be assigned to the CH_2_OH-related mode and C–O–H deformation [43]. The band at 1406 cm^−1^ could be attributed to the symmetric stretching of the carboxyl group (–COO) [6]; this band is usually found with a higher wave number, 1412 cm^−1^, but Xiao et al. [44] reported that it decreased to values around 1405 cm^−1^ after the drying of the films.

Several bands could be assigned to groups of proteins, due to the high protein content of chickpea flour (around 19 g/100 g). Thus, the 1330 cm^−1^ peak could be assigned to the Amide III mode (–NH bending, C– stretching vibration), and the 1630 and 1660 cm^-1^ bands to the Amide I mode (mainly C=O stretching vibration) [6,45,46]. Other authors related the 1630 cm^−1^ peak to water absorbed in the amorphous region of starches [3].

The bands at 2925 and 2883 cm^−1^ indicated the presence of CH_2_ groups and were related to C–H stretching [6,9]. The absorbance band at 3288 cm^−1^ corresponded to the stretching of the free, inter- and intramolecular hydroxyl groups caused by the formation of hydrogen bonds between contiguous molecules [3,39]. Similar peaks and intensities were found in films with 3% glycerol at pH 7 and 10; alkalization did not produce structural modifications in flour components.

The reduction of plasticizer content produced a pronounced effect on the FTIR spectra. Films containing 1% glycerol showed a great decrease in absorbance in all bands (Figure 1), some band shifts and several new peaks. This effect of glycerol on the FTIR spectra has been reported in gums films [46,47]. The highest concentration of plasticizer could attach along polymer chains (in this case, carbohydrates and proteins), replacing polymer–polymer interactions and preventing the forces responsible for maintaining polymer molecules together, such as hydrogen bonding, Van der Waals forces, etc. [20]. In addition, glycerol formed new bonds with polymers, other glycerol molecules and, also, with water. These interactions made films more extensible and their dry matter content decreased (Table 1 and Table 3). At low plasticizer concentration, antiplasticization may occur. This effect in films has been attributed to the reduction of polymer free volume, and to interactions between plasticizer and polymer [20]. Films containing 1% glycerol showed typical antiplasticization effects, such as a decrease in WVP and increased stiffness (Table 1 and Table 3, respectively), compared to films with high glycerol content. The drop in absorbance in these films could be related to the decrease in their water content; Warren et al. [40] reported more pronounced peaks in the FTIR spectra of hydrated starch samples compared with dry ones. The addition of a low concentration of plasticizer could also lead to an increase in polymer crystallinity and a decrease in the energy of the polymer state charge that enhances the formation of order of structures in amorphous regions [20]. Other observations of the FTIR spectra could support this explanation and may be associated with the crystallization or retrogradation of starch. As was mentioned before, the bands at 996 and 1047 cm^−1^ are related to more ordered starch structures, becoming more defined in more crystalline samples, while the band at 1020 cm^−1^ corresponds to disordered structures and increases in more amorphous samples [40]. Two ratios are commonly calculated from the absorbance of these bands to estimate the short-range ordered structure of starch; the 1047/1020 cm^−1^ ratio represents the order in more crystalline regions and the 996/1020 cm^−1^ ratio corresponds to the state of organization of the double helices localized inside crystallites, and the hydration sensitivity [40,41]. When retrogradation occurs, both ratios increase which is consistent with a more ordered structure [41]. The results of the ratio calculations are shown in Table 4. Interactions between pH and glycerol content were observed. The 1047/1020 cm^−1^ ratio was significantly higher in the 1% glycerol films than in those with 3% plasticizer, suggesting an increased organization of structure in films with low glycerol concentration; this organization was higher in these films at pH 10. A similar effect of high pH values in retrogradation has been reported in starch extracted from tortillas, produced by thermal alkaline treatment [37]. The 996/1020 cm^−1^ ratio was also higher in 1% glycerol films at alkaline pH, and is consistent with the higher retrogradation of starch in these samples. However, 1% glycerol films at neutral pH showed the lowest value for this ratio, which could be due to the lower degree of organization of the double helices and higher water sensitivity of these samples. It is possible that the interactions of starch with other components of chickpea flour, mainly proteins, could influence these results.

Both ratios were not affected by pH value in the 3% glycerol films. The interactions between plasticizer and polymer structure might prevent retrogradation.

Other characteristic peaks related to retrogradation were also observed. A band at 2854–2856 cm^−1^ only appeared in the 1% glycerol films, which overlaps the band at 2882 cm^−1^ detected in the 3% glycerol films. It has been reported that this band increases with starch retrogradation and has been attributed to the unwinding of protein or to the formation of an amylose–lipid complex [41]. Other peaks related to retrogradation (1455, 1536 and 1743 cm^−1^) were only detected in the 1% samples. These bands can be assigned to conjugated carbonyl and carboxyl groups, and C–O vibrations; the 1743 cm^−1^ band has been associated with the lipid and protein groups [37,41].

The band at 1020 cm^−1^ in the 3% glycerol films shifted towards 1030 cm^−1^ in the 1% glycerol films, probably due to the break of hydrogen bonding with water molecules [48], likely showing a weaker interaction between bound water and starch. Hydrogen bond interactions between the oxygen of the C–O–C group of starch and glycerol [49] could decrease due to the low concentration of plasticizer.

### 2.6. Thermal Stability

**Figure 2:**
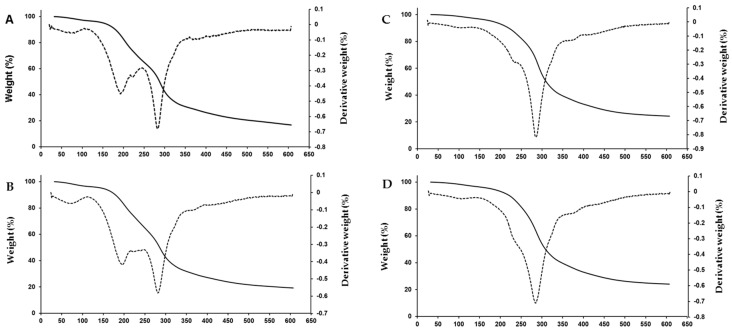
Thermogravimetric analysis (TGA) and derivative thermogravimetric (DTG) curves of chickpea flour films. (**A**) 3% glycerol, pH 7; (**B**) 3% glycerol, pH 10; (**C**) 1% glycerol, pH 7; (**D**) 1% glycerol, pH 10. 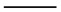 TGA; 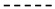 DTG.

Figure 2 shows the thermogravimetric analysis (TGA) and the first derivative of the TGA results (DTG) graphs of the thermal degradation pattern of chickpea flour films. DTG curves are derived from the raw thermogravimetric data of percentage of mass loss versus temperature. The temperature peaks, weight loss and residual weight of films are shown in Table 5.

The thermal decomposition of chickpea flour films occurred in three main weight loss stages, as has been reported for other carbohydrate-based films with glycerol as the plasticizer [9,50,51]. The first weight loss stage corresponded to the losses of free water and water linked by hydrogen bonds to film matrix. The second stage was related to the evaporation of the glycerol-rich phase, the degradation of low molecular weight protein–carbohydrate compounds and also to the evaporation of structurally bound water in the film. The third stage was associated with the decomposition of the backbone protein and carbohydrate components that remained in the films [9,38,50]. The residual mass at the end of heating, after the volatile compound removal, comprised a carbonaceous residue [52].

The glycerol content affected the thermal stability of films, while the pH of film-forming solutions did not have any effect. In the first weight loss stage, the total weight loss was similar in all films. However, the temperature peak was lower in the 3% glycerol films (80–83 °C) than in those with 1% glycerol (111–115 °C), probably due to the presence of higher amounts of free and weakly bounded water that evaporated at lower temperature. This could also affect the increase of the final temperature of the first stage from around 120 °C in films with the highest amount of glycerol to 140 °C in films with the lowest plasticizer content, in which water may have had a stronger bond to film compounds. This correlated with the lower dry matter content of the 3% glycerol films (Table 1).

In the second decomposition stage, weight loss in the 3% glycerol films was double that of the 1% glycerol films, and a temperature peak in the DTG curves at 201–204 °C was only detected in those films. This fact could be a consequence of glycerol evaporation; although pure glycerol has a boiling point of 290 °C [52], its interaction with starch and proteins in films could change its volatility. Ayala et al. [53] reported a temperature peak in this stage attributable to glycerol degradation in cassava starch biopolymers. In this stage, water strongly bound to the film structure also evaporated; the increase in weight loss in the 3% glycerol films could also be due to their higher content in chemisorbed water favored by the presence of glycerol [50]. Their higher moisture content (Table 1) could confirm this affirmation.

The highest weight loss happened in the third decomposition stage (Table 5). A peak temperature at 286–289 °C was detected in all samples. Degradation temperatures close to these have been reported by other authors in carbohydrate-based films [19,38,53,54]. Some variations in decomposition temperature could be attributed to the chemical composition and the characteristics of the crystallinity of the films [38]. The presence of high amounts of glycerol increased the weight loss extent associated with this stage, and also decreased the residual weight of films, as has been reported in other films [50,55]. This has been attributed to the interference of glycerol with the hydrocolloid molecules that form the film matrix, decreasing the heat resistance of the material.

### 2.7. Film Microstructure

**Figure 3:**
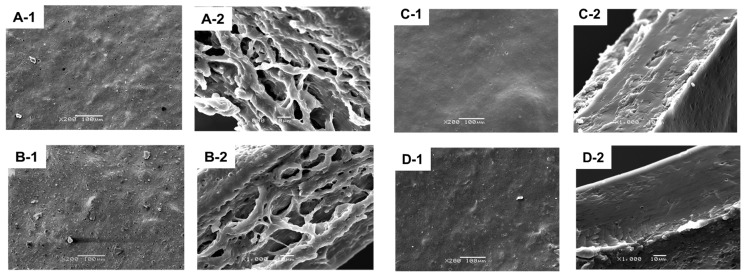
Microstructure of chickpea flour films. (**A**) 3% glycerol, pH 7; (**B**) 3% glycerol, pH 10; (**C**) 1% glycerol, pH 7; (**D**) 1% glycerol, pH 10. **1**: surface; **2**: cross section.

Figure 3 shows the SEM micrographs of the surface and a cross section of chickpea flour films. The film microstructure exhibited differences mainly related to glycerol content. Films with 3% glycerol displayed more heterogeneous surfaces (Figure 3(A-1,B-1)) than films with 1% glycerol, which showed a smoother appearance (Figure 3(C-1,D-1)). Irregularities and pores in the surface of flour film microstructures were frequently observed and could have been caused by the presence and the interaction of a mixture of macromolecules (proteins, lipids, starch and fiber) in the film matrix. This could include partial recrystallization of gelatinized starch [3,6,9] and protein aggregates. The interactions between macromolecules could also have changed due to the presence of higher amount of water in the 3% glycerol films (Table 1).

The modifications in the appearance were more intense in the film cross sections. The films with 3% plasticizer showed irregular aggregates forming a network, and a looser, more open structure (Figure 3(A-2,B-2)) than films with 1% glycerol, which showed a more dense and homogeneous appearance (Figure 3(C-2,D-2). These aggregates had some similarities with those observed in soy protein films [56]. The chickpea flour used in the film formation contained approximately 19% protein, so it is possible that proteins could form these structures in the presence of a high concentration of glycerol that modified protein molecules interaction.

The increase in interactions among macromolecules in 1% glycerol films could inhibit the formation of these structures and generate a more dense and compact, homogeneous structure without aggregates. These observations are in agreement with the lower water vapor permeability, higher opacity, tensile and puncture strength, and lower flexibility of these films compared to the films with the highest glycerol concentration (Table 1, Table 2 and Table 3). Alkalization of film-forming solutions also modified the microstructure, although to a lesser extent. At pH 10, film surfaces were rougher and cross sections more dense than those of films at neutral pH. This effect was clearer in the 1% glycerol films. Their surface showed higher amounts of granules than films at pH 7, probably due the changes in the crystallization of starch (as was observed in the FTIR results), and in protein aggregation in alkaline conditions under the direct action of the flow of air during drying. The microstructure of the 1% glycerol films was similar to that of other films based on flours [3,4,9].

## 3. Materials and Methods

### 3.1. Film Preparation

Chickpea flour, a commercial product supplied in polyethylene pouches (Intracma, Barcelona, Spain), was purchased from a local market. Total ash (gravimetrically determined), dry matter (gravimetrically determined) and crude fat contents (Soxhlet) were determined according to AACC (American Association of Cereal Chemists) International approved methods of analysis 08-16.01, 44-31.01 and 30-25.01, respectively [57,58,59]; the AACC official method 46-12.01 [60] was used for total nitrogen determination (Kjeldahl) and crude protein was calculated using a factor of 6.25. Mean values for proximate analyses of flour samples were (g/100 g): dry matter, 87.08; lipids, 6.14; protein, 19.41; ash, 3.21. These values were in the range of those reported in the literature [12].

Film forming solutions were prepared, with suspensions of chickpea flour in distilled water (6 g/100 mL water), by slow stirring for 30 min at 20 °C using a magnetic stirrer. Afterwards, glycerol (Panreac, Barcelona, Spain) was added in two proportions, 3% and 1% (*w*/*v*) (50 g glycerol/100 g flour and 16.7 g glycerol/100 g flour, respectively). Then, the pH was adjusted to 7.0 or 10.0 with 1 N NaOH. Basic pH values increased legume protein solubility [29]. The dispersions were stirred for an additional 30 min and, after, were subjected to heat treatment at 80 °C in a water bath under stirring for 20 min. Heat treatment temperature was selected close to the values of the gelatinization temperature of chickpea starch and the denaturation temperature of chickpea protein [16,61]. The solutions (0.24 g cm^−2^) were casted on leveled silicone plates (35 cm diameter) and dried at 35 °C in an air forced cabinet for 20 h. After, the plates were kept at 20 °C and 50% relative humidity for 48 h before peeling the films off. The films were stored in desiccators at 50% relative humidity for further testing. All experiments were performed in quadruplicate.

### 3.2. Film Thickness

Film thickness measurements were carried out at 36 different positions of each film selected randomly, using a 0–25 mm electronic digital micrometer (Selecta, Barcelona, Spain) with 0.001 mm resolution.

### 3.3. Water Vapor Permeability

Water vapor permeability was evaluated using the method described by Díaz et al. [62], based on the ASTM (American Society for Testing and Materials) E96-93 method [63]. Film samples were adjusted and sealed on glass cups containing dried silica gel; the diameter of the exposed area was 3.34 ± 0.12 cm. The cups were weighed and placed in desiccators with distilled water in a chamber adjusted at 20 °C. The water vapor permeability (WVP) was calculated as the weight gain of the cups due to the vapor permeated through the films and absorbed by the silica gel. Measurements were done every 2 h until constant weight. WVP was calculated using the following formula:

WVP (g mm/h m^2^ Pa) = (*w* × *L*)/(*A* × Δ*P*) (1)

where *w* is the weight gain of the cell (g) after 1 h; *L* is the film thickness (mm); *A* is the area of the exposed film (m^2^), and Δ*P* is the partial water vapor pressure difference (Pa) across the two sides of the film.

### 3.4. Dry Matter Content and Solubility

The dry matter content and solubility of the films was determined according to the methods reported by Díaz et al. [62]. The dry matter content of film samples (20 × 20 mm) was determined gravimetrically after drying in an oven (Indelab, mod. IDL.AI 80, Navarra, Spain) for 24 h at 105 °C. For solubility in water measurements, pieces of 25 × 25 mm (0.13 ± 0.03 g) of each film were used. The solubility in water of films was expressed as the percentage of film dry matter solubilized after 24 h immersion in water at 24 °C. All determinations were done in triplicate.

### 3.5. Color and Opacity

The color and opacity of films was determined according to the methods reported by Díaz et al. [62]. Color and opacity measurements were carried out in the CIE L*a*b* color space using a spectrophotometer X-Rite mod. SP60 (Grand Rapids, MI, USA) with D65 illuminant, an opening of 14 mm and the 10° standard observer. Three color measures in three random positions of each film were determined. The difference in color (ΔE*) was calculated as:

ΔE* = [(ΔL*)^2^ + (Δa*)^2^ + (Δb*)^2^]^½^. (2)

where ΔL*, Δa* and Δb* represent the differentials between the color parameter of the sample and the white tile used as background in color measurements (L* = 96.02, a* = −0.33, b* = 1.46).

### 3.6. Swelling Property

The swelling property of films was measured according to Cao et al. [64]. Air-dried film samples (2.5 × 2.5 cm) were weighted and immersed in deionized water at 25 °C for 2 min. After the removal of the liquid excess of the surface, samples were weighed. The determinations were done in triplicate. The water adsorbed by the film samples was calculated using the following equation:

Swelling (%) = [(Ww − Wd)/Wd] × 100(3)

where Ww and Wd are the weights of the wet and air dried samples, respectively.

### 3.7. DPPH Radical Scavenging Capacity

The DPPH radical scavenging capacity of the antioxidant compounds of films was determined according to the method described by Vargas et al. [8]. Film samples (1 cm^2^) were placed into assay tubes containing 3.9 mL of a 0.06 mmol/L methanolic DPPH (2,2-diphenyl-1-picrylhydrazyl; Sigma Aldrich, St. Louis, MO, USA) solution. The tubes were stored in darkness for 2 h 30 min. After film sample separation, the absorbance was measured in a spectrophometer (mod. T70+UV/Vis, PG Instruments Ltd., Lutterworth, UK) at 517 nm. Methanolic DPPH solution was used as blank and a control was prepared by the replacement of the sample with a synthetic plastic film. The determinations were done in quadruplicate. The antioxidant activity of films was calculated as the inhibition percentage of the DPPH radical (I) as follows:

I (%) = [(Ab − Af)/Ab] × 100(4)

where Ab is the absorbance of the blank and Af is the absorbance of the film.

### 3.8. Mechanical Properties

Mechanical properties were determined using a texturometer (mod. EZ Test) and the Trapezium2 Data Processing System software version 2.22E (2004), both produced by Shimadzu Corporation (Tokyo, Japan). Tensile strength, elongation at break, elastic modulus, puncture strength and puncture deformation were determined according to the ASTM D882 method [65] as described by Díaz et al. [62].

### 3.9. Fourier Transform Infrared Spectroscopy (FTIR)

The attenuated total reflectance-FTIR spectra of chickpea flour films were obtained using an ABB Bomen spectrometer mod. MB102 (Québec, QC, Canada) was in the range of 400 to 4000 cm^−1^ wavenumber. Each film spectrum was the result of 16 scans at 1 cm^−1^ resolution. Determinations were done in triplicate. Analysis of spectral data was performed with the PeakFit software version 4.12 (SYSTAT Software, Richmond, CA, USA). All spectra were base-line corrected and smoothed with a Savitsky-Golay function to remove possible noise before further data analysis. The identification of band positions was accomplished by Fourier deconvolution. Parameters of Fourier deconvolution were selected after several trials to give reasonable fits. Films spectra were deconvoluted with a Gaussian response function deconvolution filter constant of 74.9%.

### 3.10. Thermogravimetric Analysis (TGA)

Thermogravimetric analysis of dried films was carried out using a Mettler Toledo thermogravimetric analyzer with differential scanning calorimetric capability, mod. TGA/DSC1 (Schwerzenbach, Switzerland). Samples (7–9 mg) were heated in an aluminum pan from 30 to 600 °C at a rate of 10 °C/min under a nitrogen atmosphere (30 mL/min). The first derivative of the TGA curve (DTG) was calculated using the PeakFit software version 4.12 (SYSTAT Software, Richmond, CA, USA). Determinations were done in triplicate.

### 3.11. Scanning Electron Microscopy

Film microstructure was analyzed by scanning electron microscopy using a JEOL-JSM 6360LV scanning electron microscope (Jeol Ltd., Tokyo, Japan) operated at 15 kV. Sample preparation was carried out as described by Díaz et al. [62].

### 3.12. Statistical Analysis

SPSS version 19.0.0 for Windows (2010; SPSS Inc., Chicago, IL, USA) was used for data evaluation. Prior to statistical analysis, data were checked for outliers, and normal distribution was tested using the Kolmogorov-Smirnov test. Two types of analysis of variance were carried out. One-way ANOVA and the least significant difference test were used to test and compare, respectively, the statistical significance of differences among means. Two-way ANOVA was used to determine the effects of the amount of glycerol added and the pH of film-forming solutions, and their interactions, on film characteristics; General Linear Model procedure was applied. For all mean evaluations, a significance level of *p* < 0.05 was used.

## 4. Conclusions

Chickpea flour was shown to be a good base material for film preparation. The amount of glycerol added as a plasticizer and the pH of film-forming solutions have a great influence on chickpea flour film properties. Films with the lowest glycerol concentration showed a more homogeneous and dense microstructure, increased structural organization consistent with the retrogradation of starch, and higher thermal stability compared with films with the highest amount of plasticizer. These observations can be related to the lower water vapor permeability, higher opacity, mechanical resistance and rigidity, and lower flexibility. The pH of film forming solutions also affected film properties, and many interactions with glycerol content were observed. Alkalization increased thickness, swelling and greenness in all films, and the flexibility of 3% glycerol films. However, the changes were more pronounced in the 1% glycerol films: At pH 10, films were more soluble, yellow, resistant, rigid and dense than films from solutions at neutral pH. According to these findings, the pH value of film-forming solutions and the glycerol content were factors that can be adjusted to produce chickpea flour films with tailored properties that satisfy particular requirements in food applications.

## Figures and Tables

**Table 1:** Water vapor permeability, thickness, dry matter, solubility, swelling and DPPH radical scavenging capacity of films.

Film	WVP ^1^	Thickness	Dry matter	Solubility	Swelling	DPPH ^3^
		(µm)	(g/100 g)	(% D.M. ^2^)	(%)	(%)
3% glycerol						
pH 7	1.63 ± 0.11 b	177.99 ± 4.70 c	68.78 ± 0.73 a	41.89 ± 1.35 ab	82.77 ± 0.93 a	14.27 ± 0.70 d
pH 10	1.70 ± 0.11 b	187.87 ± 3.34 d	70.12 ± 0.88 b	40.04 ± 0.83 a	112.89 ± 2.74 c	13.18 ± 0.70 c
1% glycerol						
pH 7	0.83 ± 0.05 a	141.30 ± 6.30 a	88.73 ± 0.83 c	41.40 ± 1.97 a	99.49 ± 2.14 b	3.80 ± 0.22 a
pH 10	0.81 ± 0.03 a	154.79 ± 3.30 b	88.48 ± 0.57 c	43.65 ± 0.99 b	127.45 ± 3.30 d	6.66 ± 0.34 b
Analysis of variance						
Glycerol	***	***	***	*	***	***
pH	NS	***	NS	NS	***	**
Interaction	NS	NS	NS	*	NS	***

^1^ WVP—water vapor permeability expressed in g mm/kPa h m^2^; ^2^ dry matter; ^3^ DPPH—2,2-Diphenyl-1-picrylhydrazyl radical scavenging capacity. Means in the same column with different letters (a–d) are significantly different (*p* < 0.05). NS—not significant (*p* > 0.05); * *p* < 0.05; ** *p* < 0.01; *** *p* < 0.001.

**Table 2:** CIE L*a*b* color parameters, total color difference (ΔE) and opacity of chickpea flour films.

Films	L*	a*	b*	ΔE	Opacity
3% glycerol					
pH 7	88.16 ± 0.32 a	0.51 ± 0.07 d	21.46 ± 0.65 a	21.51 ± 0.71 a	23.74 ± 0.49 a
pH 10	88.02 ± 0.56 a	−1.01 ± 0.08 a	23.05 ± 0.79 ab	23.04 ± 0.94 ab	23.98 ± 0.44 a
1% glycerol					
pH 7	89.27 ± 0.35 b	−0.14 ± 0.02 c	22.49 ± 1.43 a	22.09 ± 1.46 a	26.84 ± 0.43 c
pH 10	88.15 ± 0.53 a	−0.40 ± 0.01 b	25.08 ± 2.17 b	24.88 ± 2.00 b	25.89 ± 0.15 b
Analysis of variance					
Glycerol	*	NS	*	NS	***
pH	*	***	*	**	NS
Interaction	NS	***	NS	NS	*

Means in the same column with different letters (a–d) are significantly different (*p* < 0.05). NS—not significant (*p* > 0.05); * *p* < 0.05; ** *p* < 0.01; *** *p* < 0.001.

**Table 3:** Mechanical properties of chickpea flour films.

Films	Tensile Strength at Maximum (Mpa)	Elongation at Break (%)	Elastic Modulus (N/mm)	Puncture Strength (Mpa)	Puncture
					Deformation (%)
3% glycerol					
pH 7	0.94 ± 0.07 a	18.87 ± 2.05 b	0.71 ± 0.08 a	1.41 ± 0.05 a	8.70 ± 0.33 b
pH 10	1.24 ± 0.04 a	30.91 ± 3.83 c	0.77 ± 0.07 a	1.36 ± 0.07 a	10.60 ± 0.46 c
1% glycerol					
pH 7	7.21 ± 0.12 b	6.01 ± 0.61 a	10.17 ± 0.82 b	3.70 ± 0.35 b	3.04 ± 0.61 a
pH 10	9.15 ± 0.70 c	4.87 ± 0.83 a	15.69 ± 1.37 c	3.84 ± 0.36 b	2.62 ± 0.63 a
Analysis of variance					
Glycerol	***	***	***	***	***
pH	***	***	***	NS	*
Interaction	**	***	***	NS	**

Means in the same column with different letters (a–c) are significantly different (*p* < 0.05). NS—not significant (*p* > 0.05); * *p* < 0.05; ** *p* < 0.01; *** *p* < 0.001.

**Table 4:** FTIR ratios of chickpea flour films.

Films	1047/1020 cm^−1^	996/1020 cm^−1^
	ratio	ratio
3% glycerol		
pH 7	0.64 ± 0.02 a	0.85 ± 0.01 b
pH 10	0.63 ± 0.01 a	0.87 ± 0.01 b
1% glycerol		
pH 7	0.91 ± 0.02 b	0.69 ± 0.01 a
pH 10	1.03 ± 0.01 c	0.92 ± 0.02 c
Analysis of variance		
Glycerol	***	***
pH	***	***
Interaction	***	***

Means in the same column with different letters (a–c) are significantly different (*p* < 0.05). FTIR– Fourier transform infrared spectroscopy. *** *p* < 0.001.

**Table 5:** Thermogravimetric analysis results of chickpea flour films.

Film	Weight Loss	Temperature	Stage Total	Residual
	Stage	Peak ^1^	Weight Loss (%)	Weight (%)
3% glycerol				
pH 7	1	83.34	3.33	16.83
	2	204.1	31.94	
	3	289.3	47.85	
pH 10	1	79.81	3.05	19.25
	2	201.6	31.72	
	3	286.9	45.16	
1% glycerol				
pH 7	1	111.1	2.24	24.33
	2	nd	14.88	
	3	290.5	57.82	
pH 10	1	115.3	3.23	24.11
	2	nd	15.15	
	3	287.3	57.44	

^1^ Temperature peak—value of peak in the derivative thermogram obtained from the TGA curve; nd—not detected.

## Abbreviations

DPPH	2,2-Diphenyl-1-picrylhydrazyl
DTG	First derivative of the thermogravimetric analysis results
FTIR	Fourier-transform infrared spectroscopy
SEM	Scanning electron microscopy
TGA	Thermogravimetric analysis
WVP	Water vapor permeability
